# Dual adeno-associated virus system for selective and sparse labeling of astrocytes

**DOI:** 10.4103/NRR.NRR-D-24-01607

**Published:** 2025-06-19

**Authors:** Mei Li, Zhuang Liu, Ruixi Chen, Ziyue Zhao, Qingqing Zhou, Ning Zheng, Jie Wang, Hanbing Wang

**Affiliations:** 1 Department of Anesthesiology, The First People’s Hospital of Foshan, Foshan, Guangdong Province, China; 2 National Center for Magnetic Resonance in Wuhan, Wuhan Institute of Physics and Mathematics, Innovation Academy for Precision Measurement Science and Technology, Chinese Academy of Sciences, Wuhan, Hubei Province, China; 3 University of Chinese Academy of Sciences, Beijing, China; 4 Department of Radiology, Songjiang Research Institute, Shanghai Key Laboratory of Emotions and Affective Disorders, Songjiang Hospital Affiliated to Shanghai Jiao Tong University School of Medicine, Shanghai, China; 5 Department of Anesthesiology; Brain Research Center; Zhongnan Hospital, Wuhan University, Wuhan, Hubei Province, China; 6 Clinical & Technical Support, Philips Healthcare, Wuhan, Hubei Province, China; 7 Institute of Neuroscience and Brain Diseases; Xiangyang Central Hospital, Affiliated Hospital of Hubei University of Arts and Science, Xiangyang, Hubei Province, China

**Keywords:** astrocytes, chemogenetic modulation, dual-adeno-associated virus system, glial fibrillary acidic protein (GfaABC1D) promoter, hierarchical clustering approach, morphological parameter analysis, PHP.eB, Sholl analysis, spared nerve injury, sparse labeling

## Abstract

Astrocytes are the most abundant glial cells in the central nervous system. They perform a diverse array of functions, with a critical role in structural integrity, synapse formation, and neurotransmission. These cells exhibit substantial regional heterogeneity and display variable responses to different neurological diseases. Such diversity in astrocyte morphology and function is essential for understanding both normal brain function and the underlying mechanisms of neurological disorders. To investigate this heterogeneity, we developed a novel method for the selective and sparse labeling of astrocytes in various brain regions. This technique utilizes a dual adeno-associated virus system that allows for the expression of Cre recombinase and enhanced green fluorescent protein under the control of the glial fibrillary acidic protein (GfaABC1D) promoter. The system was tested in C57BL/6J mice and successfully labeled astrocytes across multiple brain regions. The method enabled the detailed visualization of individual astrocytes—including their intricate peripheral processes—through three-dimensional reconstructions from confocal microscopy images. Furthermore, the labeling efficiency of this dual adeno-associated virus technology was validated by examining astrocyte function in a spared nerve injury model and through chemogenetic modulation. This innovative approach holds great promise for future research because it enables a more comprehensive understanding of astrocyte variation not only in spared nerve injury but also in a broad spectrum of neurological diseases. The ability to selectively label and study astrocytes in different brain regions provides a powerful tool for exploring the complexities of these essential cells and their roles in physiological and pathological conditions.

## Graphical abstract

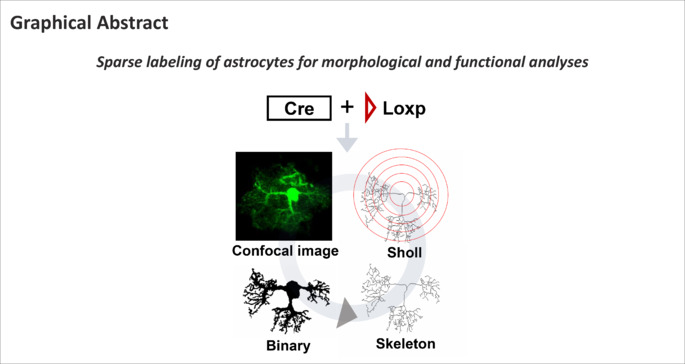

## Introduction

Astrocytes are the main type of glial cells in the central nervous system (CNS). They are both fascinating and ubiquitous, comprising approximately 20%–40% of the cellular population of the brain (Allen and Lyons, 2018; Zhou et al., 2019a). These cells engage in extensive interactions with neurons and potentially all other cell types in the CNS. Their functions include structural support, nutrient transport, neurotransmitter regulation, ion balance maintenance, neurovascular coupling, neurogenesis, synaptic remodeling, inflammatory responses, and neural circuit modulation (Giovannoni and Quintana, 2020; Oliveira and Araque, 2022; Díaz-Castro et al., 2023; Murphy-Royal et al., 2023). Astrocytes establish extensive interactions with neurons and potentially with all other types of cells in the CNS (Khakh and Sofroniew, 2015; Allen and Lyons, 2018). Age-related shrinkage of astrocytic territorial domains and a reduced volume fraction of their peripheral processes have been observed in the aging brain (Popov et al., 2021). Astrocytes in different layers exhibit distinct morphologies, which are reflected in differences in cell orientation, territorial volume, and arborization. In addition to Alzheimer’s disease risk genes that are related to morphology, there are significant associations between morphology-related genes and those associated with other common CNS disorders (Lanjakornsiripan et al., 2018; Endo et al., 2022). Recognizing the diverse morphology of astrocytes is thus crucial for understanding both normal neuronal function and disease mechanisms.

Over a century ago, Cajal et al. (1995) described the diverse shapes and structures of astrocytes in his seminal works. Astrocytes are categorized based on their morphology; distinct subtypes exhibit different structural features. Protoplasmic astrocytes, which are predominantly localized in the gray matter of the brain and spinal cord, have a soma from which multiple branches extend into surrounding neural tissue. These branches include endfeet that contact blood vessels as well as branchlets and leaflets that contribute to the tripartite synapse (Tabata, 2015; Hayden, 2023). In adult mice, the mean volume of an astrocyte in the hippocampus (HIP) is 3.8 × 10^4^ μm^3^, with over 90% of its surface area consisting of branches, branchlets, and leaflets (Zhou et al., 2019a). By contrast, fibrous astrocytes are more common in the white matter of the CNS, particularly around myelinated nerve fibers. These cells are characterized by numerous fibrils in their cytoplasm. Their primary processes extend radially from the soma, forming expansions and endfeet that contact vascular capillary surfaces (Köhler et al., 2021; Verkhratsky et al., 2021). Specialized astroglial cells also exist, such as Müller cells in the retina and Bergmann astrocytes in the cerebellum (De Zeeuw and Hoogland, 2015; Vecino et al., 2016; Yoo et al., 2021). Bergmann astrocytes have long, fibrous processes that extend through the molecular layer to the cerebellar surface, running parallel to the dendrites of Purkinje cells. These extensions play a key role in the structural organization and function of the cerebellum (Olude et al., 2015).

When studying the morphology of astrocytes, it is crucial to accurately assess and monitor alterations in their structure. However, existing methods for analyzing astrocyte morphology have certain limitations. For example, the use of immunohistochemistry to visualize glial fibrillary acidic protein (GFAP) significantly underrepresents astrocyte complexity and fails to detect many astrocytes (Sofroniew and Vinters, 2010; Reeves et al., 2011). An alternative method of visualizing astrocytes in brain sections involves briefly exposing them to fixative before introducing fluorescent dyes via a sharp electrode, using an iontophoresis-based technique (Bushong et al., 2002). This technique allows dye to spread throughout the cell, thereby revealing the complete morphology of an astrocyte. Moreover, this method can be easily integrated with immunohistochemistry to enable the confirmation of cell type and the examination of protein expression across different cell compartments (Moye et al., 2019; Zhou et al., 2019a). However, filling cells with intracellular dye is a time-intensive process that allows for the concurrent sampling of only a few cells. For *in vivo* sparse labeling conducive to single-cell morphological analysis, reporter expression via viral vectors or transgenic mouse lines has been used. This approach includes the expression of cytosolic fluorescent proteins (e.g., green fluorescent protein [GFP] and tdTomato) and membrane-bound fluorescent proteins (e.g., Lck-GFP) driven by astrocyte-specific promoters. Mouse strains engineered to express fluorescent reporters under the control of astrocyte-specific promoters enable the high-definition imaging of these cells. In one study, astrocytes were marked with enhanced GFP (EGFP) using mice that express the tetracycline-controlled transactivator protein under the human GFAP promoter (GFAP-tTA mice). The researchers observed that individual cortical astrocytes typically encompass approximately four neuronal somata and engage with 300–600 neuronal dendrites. Additionally, Lck-GFP has been used to study the effects of brain-derived neurotrophic factor on the morphological characteristics of astrocytes (Holt et al., 2019).

Although diffraction-limited light microscopy is commonly used for studying astrocytes, it is inadequate for discerning how branchlets and leaflets sprout from larger branches or for precisely quantifying their numbers or sizes, meaning that the structural organization of astrocyte morphology remains unclear. In the present study, to overcome these limitations and observe astrocyte morphology using light microscopy, we developed a dual adeno-associated virus (AAV) system (DAS) that achieved sparse, bright labeling of single astrocytes. Our system included one AAV vector containing a GFAP (GfaABC1D) promoter-driven Cre expression cassette, and another AAV vector containing a GfaABC1D promoter followed by a Cre-dependent EGFP cassette. A particular advantage of our DAS was its ability to sparsely label astrocytes. When a solution with an appropriate ratio between the two viruses was injected into the brain, several metrics for assessing single astrocytes were able to be imaged using light microscopy, thereby allowing us to quantify territories, branching patterns, neuropil infiltration volumes, and overall territory volumes. This approach is advantageous for detailed morphological assessments, providing a clear understanding of the complex structural organization of astrocytes. The ability to selectively label and study astrocytes in different brain regions provides a powerful tool for revealing the complexities of these essential cells and their roles in health and disease.

## Methods

### Cells and plasmids

Because HEK293 cells have high plasmid transfection efficiency, a well-established protocol, and are suitable for packaging various AAV serotypes, we performed the packaging of the rAAV three-plasmid system in HEK293 cells (ATCC, Manassas, VA, USA, Cat# CRL-1573, RRID: CVCL_0045). The cells were maintained in Balance CD293 culture (CELL-WISE, Shanghai, China, Cat# CW01001) supplemented with 1% penicillin-streptomycin (Gibco, Darmstadt, Germany, Cat# 15140122) at 37°C and 5% CO_2_. The pAAV-GfaABC1D-Cre-SV40 and pAAV-GfaABC1D-DIO-EGFP plasmids were provided by BrainCase (Shenzhen, China).

### Recombinant adeno-associated virus vector production and virus titration

Recombinant AAV (rAAV) virions were produced in HEK293 cells using a conventional triple-plasmid transfection method (Kimura et al., 2019). Briefly, HEK293 cells were seeded at 80% confluence and co-transfected with pAAV-Cap, pAAV-GfaABC1D-Cre or pAAV-GfaABC1D-DIO-EGFP, and pAAV-helper plasmids using polyethylenimine reagent (Polyscience, Warrington, USA, CAS No. 49553-93-7). Following a 72-hour iodixanol gradient centrifugation, as previously outlined (Liu et al., 2024), the titers of the purified AAV virions were quantified using quantitative polymerase chain reaction (qPCR) with SYBR Green PCR Master Mix (Bio-Rad, CA, USA, CAS No. 1725270) and specific primer pairs targeting the woodchuck hepatitis virus sequence: forward 5′-TCC CAT AGT AAC GCC AAT AGG-3′, reverse 5′-CTT GGC ATA TGA TAC ACT TGA TG-3′. Standard curves were generated through 10-fold serial dilutions of standard plasmids. Additionally, titers of AAV pseudo-vectors and capsid compositions of AAV vectors were evaluated using silver staining analysis (Liu et al., 2024). Briefly, 10 μL AAV vector sample (approximate concentration: 1 × 10^12^ viral particles/mL) was subjected to electrophoresis on a 10% sodium dodecyl sulfate polyacrylamide gel (Vazyme, Nanjing, China, CAS Number: E303-01), followed by standard silver staining procedures as described by the manufacturer (Thermo Fisher Scientific, Waltham, MA, USA, CAS No. 24612). We purified the self-packaged viruses and determined their titers in these experiments. The DAS relied on Cre recombinase, which catalyzed site-specific recombination at loxP sites flanking the gene of interest. The EGFP marker gene was inserted in an inverted orientation between two loxP sites, creating a double-floxed inverse open reading frame (DIO). In the presence of Cre recombinase, the loxP sites were recognized, and the DNA segment between them was excised, thus flipping the marker gene into the correct orientation for expression. The viruses of rAAV2/5-GfaABC1D-mCherry, the rAAV2/5-GfaABC1D-DIO-mCherry virus and rAAV2/5-GfaABC1D-Cre were provided by BrainCase (Shenzhen, China).

### Animals

Eight-week-old male C57BL/6J mice weighing approximately 25–28 g (Rechlin et al., 2022) were housed in a temperature-controlled room with an ambient temperature of 22 ± 2°C and a 12-hour light/12-hour dark cycle to mimic natural day–night rhythms. Animals were kept in ventilated, pathogen-free cages with no more than five mice per cage to prevent overcrowding and stress. Mice had *ad libitum* access to a standardized, nutritionally complete rodent diet, formulated to meet the nutritional needs of laboratory mice. This study was performed under the National Institutes of Health Guide for the Care and Use of Laboratory Animals (8^th^ ed., National Research Council, 2011) to ensure the humane treatment of animals. All procedures were approved by the Animal Care and Use Committee at the Guangdong Medical Laboratory Animal Center, China (C202303-6). The animals used for all experiments were naive and had undergone no previous experimental use or drug tests. They were purchased from the Guangdong Medical Laboratory Animal Center, China (license No. SCXK (Yue) 2022-0002). The animals had a specific genetic background and genotype and were of specific-pathogen-free grade. Their health and immune status were monitored, and no drug treatments were administered prior to the experiments. For all procedures, anesthesia was induced using sodium pentobarbital (intraperitoneal injection). The groups were assigned as follows: the control (Con) group (mice received stereotaxic injections of one type of vector) and the DAS group (mice received stereotaxic injections of the same dose of AAV-Cre and AAV-DIO vectors).

### Animal surgery for virus infection

#### Stereotaxic injections

C57BL/6J mice were deeply anesthetized using sodium pentobarbital (50 mg/kg body weight; Sigma-Aldrich, St. Louis, MO, USA, Cat# 76229) administered via intraperitoneal injection. The anesthetized mice were then placed in a stereotaxic frame (RWD, Shenzhen, China, 68025 - stereotaxic apparatus and 68030 - mice adaptor). A small hole was drilled through the skull at the site aligned with the target coordinates. The coordinates for the injection site were determined based on the Mouse Brain Atlas (Paxinos and Franklin, 2013). After exposing the skull, a microdrill was used to create a small opening, and the dura mater was carefully punctured to minimize tissue damage. A syringe attached to a micropump was used to inject the substance at a controlled rate, thereby ensuring precise delivery to the target area (volume: 100 µL; titer: 1 × 10^11^ viral genomes [vg]/mouse). After the injection, the needle was left in the injection site for 15 minutes to allow the substance to diffuse away from the injection site and reduce backflow along the needle track. The incision site was then cleaned and the skin was sutured. Subsequently, animals were returned to their recovery cages and monitored for signs of distress or infection.

#### Tail vein catheterization for PHP.eB viruses

The mouse tail vein was warmed using warm water, and the tail vein was enlarged for needle catheterization. After catheterization, the PHP.eB virus (total volume: 100 μL of viral solution) was slowly injected into the tail vein. The viral solution of rAAV/PHP.eB-GfaABC1D-EGFP was 1 × 10^11^ vg/mouse, and the viral solution of rAAV/PHP.eB-GfaABC1D-DIO-EGFP in the system was 1 × 10^11^ vg/mouse. The rAAV/PHP.eB-GfaABC1D-Cre doses were adjusted according to the virus ratio: rAAV/PHP.eB-GfaABC1D-Cre:rAAV/PHP.eB-GfaABC1D-DIO-EGFP = 1:1 (DIO/cre=1:1 Group) or 1:10 (DIO/cre=1:10 Group). After the injection, the needle was kept in the tail vein for approximately 5 minutes and then withdrawn. Gentle pressure was applied to the injection site with gauze to prevent bleeding. The mice were then allowed to recover.

### Microscopic analysis of brain tissue

After the experimental procedures, each animal was euthanized using anesthesia induced by intraperitoneal sodium pentobarbital (100 mg/kg body weight), and the brain was carefully removed. The brain was immersed in 4% paraformaldehyde solution overnight at 4°C. After fixation, the brain was dehydrated in a 30% sucrose solution until it sank. The brain was then embedded in optimum cutting temperature compound and sectioned into 40-μm-thick slices using a cryostat (Thermo Fisher Scientific, NX 50). The sections were stored in a cryoprotectant solution at –20°C for long-term storage or immediately processed for staining.

For staining, sections were washed three times with phosphate-buffered saline (PBS) for 5 minutes, and then blocked with blocking buffer (5% fetal bovine serum and 0.3% Triton X-100 in PBS) for 1 hour. Next, the sections were incubated overnight at 4°C with anti-GFAP antibody (goat, 1:1000; Abcam, Cambridge, UK, Cat# ab53554, RRID: AB_880202), anti-S100β antibody (rabbit, 1:1000; Abcam, Cat# ab52642, RRID: AB_882426), or anti-neuron-specific nuclear protein (NeuN) antibody (mouse, 1:1000; Abcam, Cat# ab104224, RRID: AB_10711040), followed by incubation with Cy3-labeled donkey anti-rabbit immunoglobulin G (1:500; Jackson ImmunoResearch, West Grove, PA, USA, Cat# 711-165-152) in PBS–3% (weight/volume) bovine serum albumin for 1 hour in the dark at room temperature. Nuclei were stained with 4′,6-diamidino-2-phenylindole (DAPI; Beyotime, Shanghai, China, C1006). Samples were fixed and mounted using an antifade mounting medium (Abcam, Cat# ab104135) to preserve fluorescent signals and minimize photobleaching. Stained cells were imaged under a confocal microscope (Leica Microsystems, Buffalo Grove, IL, USA, TCS SP8) or a virtual microscopy slide-scanning system (Olympus, Tokyo, Japan, VS 120). To obtain high-resolution images of astrocyte morphology, confocal microscopy was performed using a laser scanning confocal microscope (Leica TCS SP8) equipped with a 63× oil immersion objective (HC PL APO 63×/1.40 OIL). EGFP-labeled astrocytes were excited using a 488-nm laser. Leica immersion oil was used as the imaging medium. Z-stack scanning was performed to capture the three-dimensional (3D) structure of astrocytes, with an optical section thickness of 0.5–1 μm and a resolution of 10^24^ × 10^24^ pixels. Laser power and gain were optimized to achieve high signal-to-noise ratios while avoiding signal saturation. Images were saved and exported as .tiff files for further analysis. Post-acquisition processing, including 3D reconstruction and fluorescence intensity analysis, was performed using ImageJ software (version 1.53, National Institutes of Health, Bethesda, MD, USA).

### Morphological parameter hierarchical clustering

We conducted a comprehensive morphological analysis of astrocytes across different brain regions using confocal microscopy and advanced image processing techniques. Tissue sections expressing mCherry or EGFP in astrocytes, mediated by viral vectors, were imaged. Cell counting was performed on maximum intensity projections using ImageJ software (version 1.53). Morphological parameters, including signal area and mean signal intensity for astrocyte markers, were extracted and used to calculate scaled RGB scores, thereby providing a normalized measure of astrocyte marker expression.

For a more detailed structural analysis, we converted skeletonized images into graphs using graph-theoretical approaches, thereby allowing Python (version 3.11.7, Python Software Foundation, Wilmington, MA, USA) to calculate various morphological parameters such as diameter, territory size, circularity, roundness, and graph density. To visualize the differences in astrocyte morphology across brain regions, we used hierarchical clustering and generated heatmaps of the log-transformed Euclidean distances between samples. This analysis highlighted the structural variations in astrocytes across different brain regions.

### Behavioral assessment

The elevated plus maze apparatus was used to assess anxiety-like behavior in mice. The apparatus consisted of two perpendicular arms (74 cm × 5 cm) elevated 50 cm above the floor. One arm was enclosed with 14-cm-high walls (closed arm), whereas the other arm remained open without walls (open arm). Prior to testing, each mouse was placed in the center zone of the maze, facing an open arm, and was allowed to freely explore for 10 minutes. The time spent and the path taken in the open arm were recorded and analyzed using ANY-maze software (Stoelting Co., Wood Dale, IL, USA). Mice were divided into two experimental groups: the saline group (*n* = 8), which received an intraperitoneal injection of saline, and the clozapine N-oxide (CNO) group (*n* = 8), which received an intraperitoneal injection of CNO (MCE, Monmouth Junction, NJ, USA, Cat# HY-17366). Both groups were administered a dose of 3 mg/kg. Following each test, the maze was thoroughly cleaned with 75% ethanol to remove any residual odors and maintain consistent conditions for subsequent trials. The behavioral data collected from each group were analyzed to evaluate differences in anxiety-like behavior, focusing on the time spent in the open arm and the total distance traveled.

### Sholl analysis

Astrocytes were mapped in three dimensions using EGFP staining datasets and the Simple Neurite Tracer plug-in for ImageJ (Tavares et al., 2017). This method generated a skeletal representation of the astrocyte processes, identifying the locations of somas on the traced images. The traces from Simple Neurite Tracer were then used for Sholl analysis. Image datasets were read and converted to binary images and skeletonized with the skeletonize function using skimage. Sholl analysis involved calculating the intersections of astrocyte processes with concentric spheres spaced 5 µm apart; distances were computed using numpy. A graphical interface was used to manually select the root point (soma) on the skeletonized image, and the selected points were saved and loaded for future reference using json.

Visualization of the Sholl analysis was performed using matplotlib (an open-source software library for Python (version 3.11.7, https://www.python.org/downloads/); plots were created to illustrate the number of intersections as a function of radius.

### Statistical analysis

No statistical methods were used to predetermine sample sizes; however, our sample sizes were similar to those reported in previous publications (Festing and Altman, 2002; Biau et al., 2008). Statistical details for each experiment are provided in the figure legends, with “*n*” representing the number of animals per group. Significance was defined as *P* < 0.05. Statistical analyses were performed using a two-sample *t*-test or one-way analysis of variance with Bonferroni correction, as appropriate for the data type. Data are expressed as the mean ± standard error of the mean. Statistical analyses were conducted using GraphPad Prism (Version 9, GraphPad Software, Boston, MA, USA, www.graphpad.com) and Python (version 3.11.7).

## Results

### Image acquisition for the sparse labeled astrocytes

**Figure 1:**
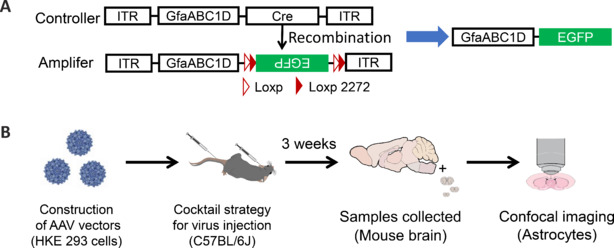
Flowcharts of the experimental design. (A) The DAS is a precision tool for astrocyte morphology analysis. (B) Schematic of the experimental procedure from virus construction to data collection. Virus injection: Stereotaxic injection or tail vein injection. AAV: Adeno-associated viruses; DAS: dual-AAV system; EGFP: enhanced green fluorescent protein; ITR: inverted terminal repeat.

The DAS used the specific GfaABC1D promoter to drive expression of the fluorescent marker EGFP in target astrocytes (**Figure 1A**). With the GfaABC1D promoter driving Cre recombinase expression, this system selectively activated the fluorescent marker in astrocytes. To assess efficiency of the DAS compared with that of the traditional GfaABC1D-EGFP virus, the PHP.eB system was used to package both viral constructs for effective blood–brain barrier penetration. The GfaABC1D-EGFP virus was administered at a dose of 100 µL with a viral titer of 1 × 10^11^ vg/mouse (**Additional Figure 1A**). The DAS used different ratios of GfaABC1D-Cre and GfaABC1D-DIO viruses, with a total viral volume of 100 µL. The GfaABC1D-DIO virus had a titer of 1 × 10^11^ particles per mouse, whereas the Cre virus titer was adjusted based on the ratio to the GfaABC1D-DIO virus (**Additional Figure 1B** and **C**). After 3 weeks of tail vein injections, mice were euthanized and brain tissue was sectioned for confocal microscopy. Labeling and imaging conditions were standardized across experiments. In various brain regions, the fluorescence intensity per cell labeled with the DAS was significantly higher than that labeled with the GfaABC1D-EGFP virus alone (**Additional Figure 1D** and **E**). Notably, the DAS using a 1:10 ratio of GfaABC1D-Cre to GfaABC1D-DIO viruses provided the distinct labeling of individual astrocytes with sparse fluorescence, thereby enabling the clear visualization of their morphological structures. In summary, the DAS demonstrated better astrocyte labeling efficiency than the traditional GfaABC1D-EGFP virus, and the 1:10 ratio of GfaABC1D-Cre to GfaABC1D-DIO viruses offered optimal results for the clear visualization of astrocyte morphology.

**Figure 2:**
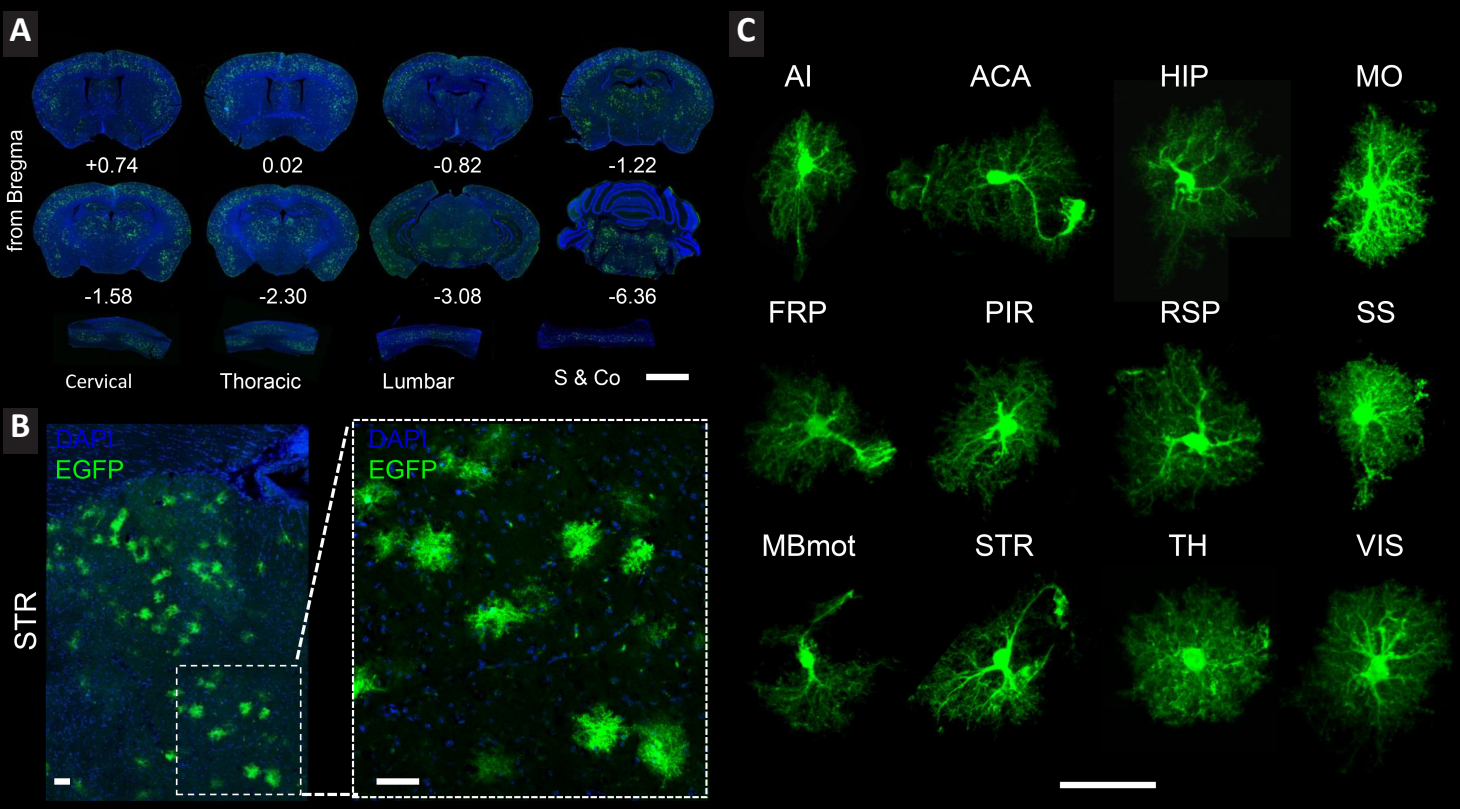
Sparsely labeled astrocytes across multiple brain regions. (A) Collection of coronal brain and spinal cord sections with stereotaxic coordinates (in mm) from bregma. The sections include cervical, thoracic, and lumbar regions as well as the S & Co segments. (B) Representative images of AAV vector-mediated expression of EGFP in the brain. The sections were counterstained with DAPI. The image on the right provides a detailed view of individual astrocytes. (C) Representative single-cell confocal images of astrocytes from various CNS regions. The DAS used a 1:10 ratio of GfaABC1D-Cre to GfaABC1D-DIO viruses. Scale bars: 2 mm in A, 50 μm in B and C. *n* = 4. AAV: Adeno-associated viruses; ACA: anterior cingulate area; AI: agranular insular cortex; CNS: central nervous system; DAPI: 4′,6-diamidino-2-phenylindole; DAS: dual-AAV system; EGFP: enhanced green fluorescent protein; FRP: frontal pole; HIP: hippocampus; MBmot: midbrain motor nucleus; MO: motor cortex; PIR: piriform cortex; RSP: retrosplenial cortex; S & Co: sacral and coccygeal; SS: somatosensory cortex; STR: striatum; TH: thalamus; VIS: visual cortex.

EGFP expression was observed in astrocytes of C57BL/6J mice following DAS injection (**Figure 1B**). Coronal sections of the brain and spinal cord (**Figure 2A**) revealed the spatial distribution of these labeled astrocytes across multiple regions, from cervical to lumbar segments (precise anatomical locations are indicated by stereotaxic coordinates). In the striatum (STR), astrocytes were clearly labeled with EGFP and counterstained with DAPI to highlight cell nuclei. An image at 25× magnification revealed the detailed morphological features of individual astrocytes and their extensive network of processes. Moreover, higher magnification (63×) confocal images from various CNS regions further illustrated the substantial morphological diversity among astrocytes, highlighting their intricate branching patterns in particular (**Figure 2B**).

To comprehensively examine astrocyte morphology across different brain regions, the brain was divided into 12 core areas, and astrocytes were analyzed using 3D reconstruction technology. Astrocytes in the agranular insular cortex (AI), anterior cingulate area (ACA), HIP, motor cortex (MO), frontal pole (FRP), piriform cortex (PIR), retrosplenial cortex (RSP), somatosensory cortex (SS), midbrain motor nucleus (MBmot), STR, thalamus (TH), and visual cortex (VIS) exhibited distinct structural characteristics in 3D overlay projections (**Figure 2C**). These characteristics included extensive branching and complex network formations. Notable variations were observed between regions, such as terminal expansions at the ends of major branches and near blood vessels or neuronal axons in the ACA and STR. These structures—key components of the blood–brain barrier—are involved in signal transmission and substance transport between astrocytes and neurons (Mishra et al., 2016; Di Giovanna et al., 2018). Additionally, cells in the MBmot region exhibited distinct polarity, suggesting the possibility of specialized cellular structures related to region-specific functions (Zhou et al., 2019a; Yu et al., 2020). Together, these findings highlight the diverse morphologies and intricate distributions of astrocytes across the CNS, emphasizing their heterogeneity and complexity.

### Region-by-brain presentation and statistics of specific markers of astrocytes

**Figure 3:**
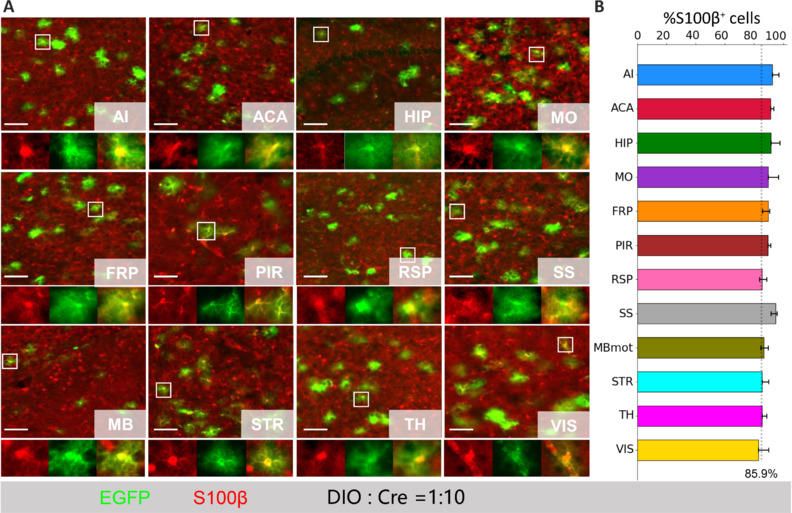
Presentation and collections of specific astrocyte markers. (A) Immunofluorescent images of astrocytes labeled with S100β (red) and EGFP (green) in various CNS regions. The inset subfigures indicate individual cells and document the co-expression of S100β and EGFP. Scale bars: 50 μm. (B) The percentage of S100β-positive cells in each CNS region using DAS technology. Data are expressed as mean ± SEM (*n* = 4). ACA: Anterior cingulate area; AI: agranular insular cortex; CNS: central nervous system; DAS: dual-AAV system; EGFP: enhanced green fluorescent protein; FRP: frontal pole; HIP: hippocampus; MBmot: midbrain motor nucleus; MO: motor cortex; PIR: piriform cortex; RSP: retrosplenial cortex; SS: somatosensory cortex; STR: striatum; TH: thalamus; VIS: visual cortex.

S100β was used as a marker for astrocyte staining because of its high specificity across a broad range of brain regions (Brozzi et al., 2009; Michetti et al., 2019). By contrast, GFAP demonstrates lower labeling efficiency in specific cortical areas. However, no single marker is ideal for all astrocyte studies (Yang and Wang, 2015). Astrocytes across different CNS regions exhibited distinct labeling patterns with S100β (red) and EGFP (green). The extensive co-expression of S100β and EGFP in astrocytes confirmed their successful labeling across various brain regions (**Figure 3A**). Additionally, NeuN and EGFP co-expression was analyzed to rule out viral leakage under the GfaABC1D promoter (**Additional Figure 2A**).

To assess the specificity of the labeling process, we quantified the percentage of S100β-positive cells in each CNS region (**Figure 3B** and **Additional Figure 2B**). The SS showed the highest percentage of S100β-positive cells, with more than 93%, followed by the AI, ACA, HIP, and MO. Other regions, such as the FRP, PIR, and RSP, also demonstrated substantial S100β positivity. The SS had the highest percentage of S100β-positive cells, whereas the MBmot had the lowest. The STR, TH, and VIS showed moderate to high percentages of S100β-positive cells, further supporting the high specificity of the DAS for astrocyte labeling (all regions exceeded 85.9%). Our results also revealed minimal viral leakage under the GFAP promoter (less than 5%), confirming the widespread presence of labeled astrocytes throughout the CNS (Baldwin et al., 2023).

### Single astrocyte morphological parameter analysis

**Figure 4:**
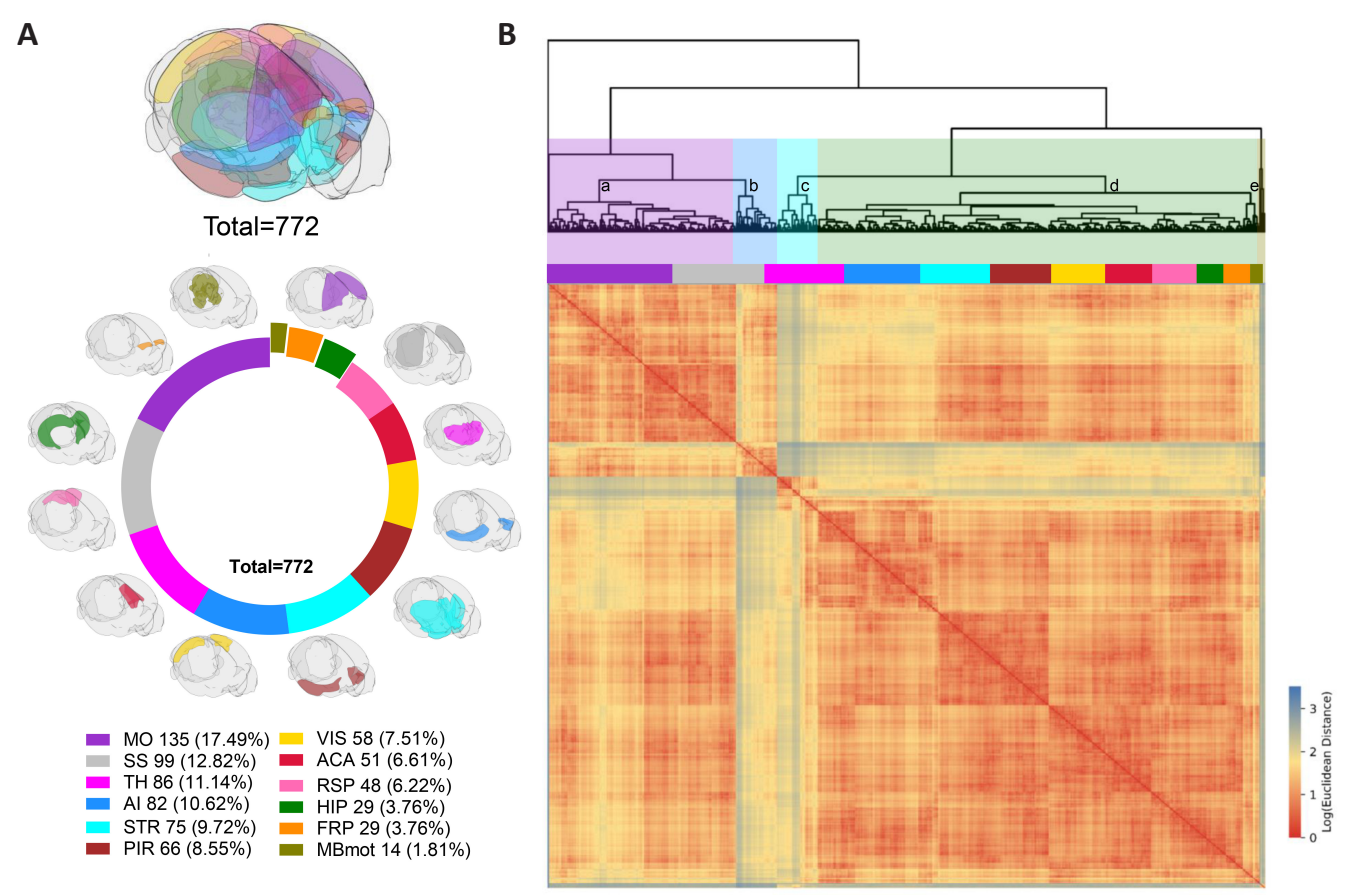
Morphological parameter analysis for single astrocytes in different brain regions. (A) Distribution and quantification of labeled astrocytes across various CNS regions. (B) Hierarchical clustering analysis of the labeled cells based on the morphological data of the astrocytes. ACA: Anterior cingulate area; AI: agranular insular cortex; CNS: central nervous system; FRP: frontal pole; HIP: hippocampus; MBmot: midbrain motor nucleus; MO: motor cortex; PIR: piriform cortex; RSP: retrosplenial cortex; SS: somatosensory cortex; STR: striatum; TH: thalamus; VIS: visual cortex.

Using DAS labeling technology, astrocytes in 12 different brain regions were observed following tail vein catheterization with PHP.eB virus injection. The anatomical locations of these regions in the brain are summarized in **Figure 4A**. Finally, 772 labeled astrocyte cells were identified and imaged from confocal images across these CNS regions. The MO exhibited the highest astrocyte concentration (135 cells; 17.49%), followed by the SS (99 cells; 12.82%), TH (88 cells; 11.14%); AI (82 cells; 10.62%), STR (75 cells; 9.72%), PIR (68 cells; 8.55%), VIS (58 cells; 7.51%), ACA (51 cells; 6.61%), RSP (48 cells; 6.22%), HIP (29 cells; 3.76%), FRP (29 cells; 3.76%), and MBmot (14 cells; 1.81%). In summary, our DAS labeling technology enabled the identification and imaging of astrocytes across 12 different brain regions, providing insights into the regional distributions and morphological diversity of these cells.

To further explore astrocyte morphology across different brain regions, we expanded the analysis to include all 772 cells. Raw images were processed using binarization and segmentation techniques (**Additional Figure 3**), followed by skeletal extraction. Morphological features, including parameters such as maximum and minimum diameters, territory sizes, circularity, and roundness, were quantified (**Additional Figure 4**). Additionally, we assessed graph-based features, including the numbers of nodes and edges, average degree, and graph density, to gain deeper insights into astrocyte structure. Hierarchical clustering was applied to identify distinct morphological patterns (**Figure 4B**). The heatmap and clustering analysis revealed unique morphological characteristics, with the correlation matrix highlighting varying degrees of similarity; red areas indicate high correlation, whereas blue areas represent low correlation. Clustering clearly delineated five distinct groups based on morphological features. Astrocytes in the MO formed a cohesive cluster, which suggests consistent structural characteristics, aligning with the results of prior studies (Clavreul et al., 2022; Endo et al., 2022). By contrast, cells from the SS and TH spanned multiple clusters, reflecting greater morphological diversity. Most cells from other brain regions fell into a general cluster (“d”), illustrating both shared and distinct features across CNS regions. Collectively, these findings underscore the structural diversity of astrocytes in the CNS and validate the effectiveness of our labeling methods for enabling their detailed morphological analysis.

### Morphological parameter analysis of astrocytes

**Figure 5:**
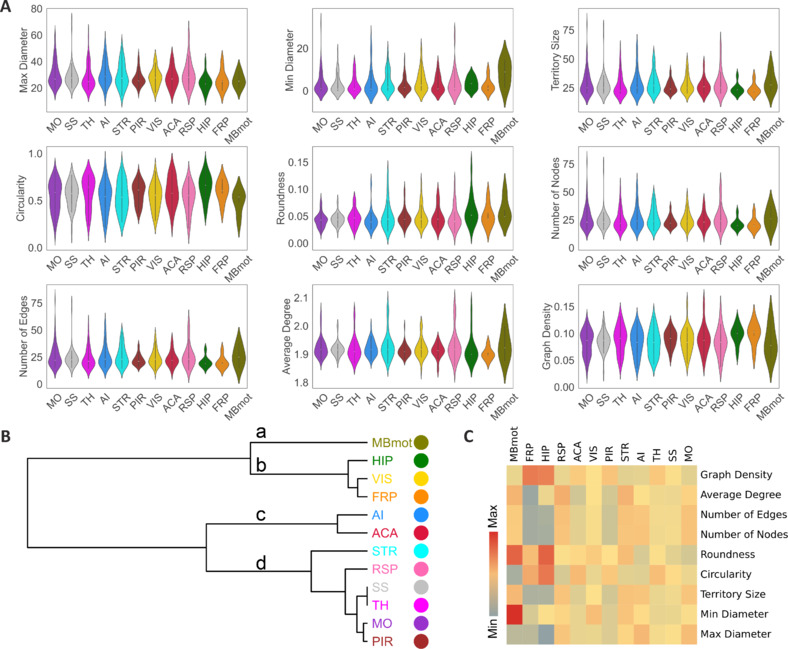
Morphological parameter analysis for labeled astrocytes in different brain regions. (A) The detailed distributions of nine morphological parameters of astrocytes across various CNS regions. (B) Results of the clustering analysis for the morphological parameters of astrocytes in the 12 anatomical regions. (C) Heatmap showing the normalized values of various morphological parameters of astrocytes across different CNS regions. Data were represented with mean ± SEM (*n* = 4). ACA: Anterior cingulate area; AI: agranular insular cortex; CNS: central nervous system; FRP: frontal pole; HIP: hippocampus; MBmot: midbrain motor nucleus; MO: motor cortex; PIR: piriform cortex; RSP: retrosplenial cortex; SS: somatosensory cortex; STR: striatum; TH: thalamus; VIS: visual cortex.

After clustering the morphological parameters of individual astrocytes, a comprehensive statistical analysis was performed to assess these parameters across the 12 brain regions, thereby providing insights into region-specific structural features. **Figure 5A** illustrates the distribution of individual morphological parameters across the CNS regions. The maximum diameter, representing the largest extent of astrocyte processes, was notably higher in regions such as the MO, SS, AI, and RSP. The minimum diameter, reflecting the smallest extent of astrocyte processes, remained relatively consistent across regions but was observed to be slightly higher in the MO. Territory size, which indicates the spatial coverage of astrocyte processes, showed significant variation, with larger territories observed in the MO and SS (both exhibiting similar distributions). Circularity and roundness, which describe the overall shape of astrocytes, were largely uniform across regions, although minor differences were noted in the MO and SS. In summary, the statistical analysis of morphological parameters across 12 brain regions revealed significant regional variations in astrocyte structure, highlighting differences in branching complexity, territorial coverage, and network density; regions such as the MO and SS exhibited larger and more intricate astrocyte networks.

The numbers of nodes and edges, indicating the complexity of astrocyte branching, were slightly higher in the MO and SS, suggesting more intricate branching in these cortical areas. The average degree, which measures the connectivity of astrocyte processes, was highest in the HIP. The SS and TH were grouped into the same category, reflecting their high morphological variability (as observed in the clustering analysis; **Figure 4B**). These results further confirmed the reliability of the single-cell morphological clustering. Graph density, reflecting the overall density of astrocyte networks, revealed higher values in regions such as the VIS and ACA, suggesting more compact and interconnected networks. The complex graph density of these regions may relate to their advanced functional roles (Cheng et al., 2019; Yu et al., 2020; Torres-Ceja and Olsen, 2022).

**Figure 5B** highlights the distinct patterns of astrocyte morphology across various CNS regions, with anatomical clustering revealing clear insights into these patterns. Regions exhibited notable differences in the numbers of nodes and edges, average degree, and graph density. **Figure 5C** shows a color scale ranging from blue (low values) to red (high values), which visually represents the relative magnitude of each morphological parameter within each region. These findings underscore the morphological diversity of astrocytes across the CNS, highlighting the complexity and variability of astrocyte structure and connectivity. Together, these results emphasize the potential for the combined analysis of multiple morphological parameters and their functional correlations (Endo et al., 2022).

### Sholl analysis based on anatomical regions

**Figure 6:**
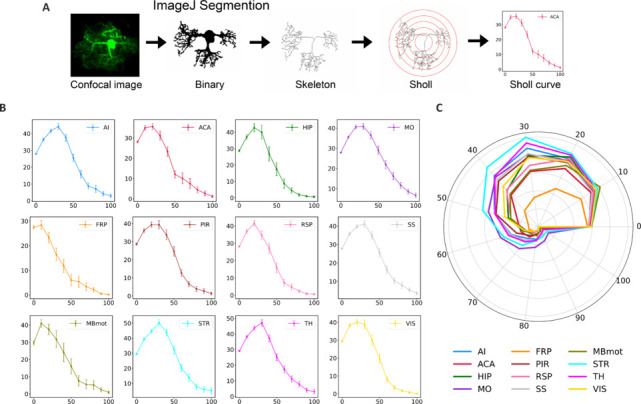
Morphological parameter analysis for the astrocytes in different brain regions. (A) Workflow of the Sholl analysis for astrocytes. (B) Sholl analysis curves for astrocytes in various CNS regions. (C) Radar plot summarizing the morphological parameters of astrocytes in different CNS regions. Data are expressed as mean ± SEM (*n* = 4). ACA: Anterior cingulate area; AI: agranular insular cortex; CNS: central nervous system; FRP: frontal pole; HIP: hippocampus; MBmot: midbrain motor nucleus; MO: motor cortex; PIR: piriform cortex; RSP: retrosplenial cortex; SS: somatosensory cortex; STR: striatum; TH: thalamus; VIS: visual cortex.

To further assess the morphological characteristics of astrocytes, we performed Sholl analysis on astrocytes from various brain regions. First, the collected images were enhanced using ImageJ. Segmentation algorithms were applied to the raw images, followed by a detailed review of the segmentation results. Binary and skeletal extractions were then performed on the segmented cell images (**Figure 6A**). This preprocessing step ensured the accuracy and reliability of the subsequent analysis.

Our analysis revealed that astrocytes from different CNS regions exhibited distinct branching complexities (**Figure 6B**). The most complex branching nodes were concentrated around 25 µm, with the FRP displaying the most intricate branching within the smallest diameter range. The highest branching complexity was observed in the STR, aligning with its clustering in **Figure 5B**. These findings suggest that branching complexity varies markedly across regions, with some areas exhibiting more intricate structures.

The detailed Sholl analysis characteristics for different regions were as follows. Astrocytes in the AI peaked at approximately 30 µm with high intersection counts, indicating moderate branching. The ACA and HIP showed similar profiles, with slightly lower peaks. Astrocytes in the MO exhibited a broader distribution, suggesting more extensive branching. The FRP and PIR showed intermediate profiles, whereas the RSP and SS displayed moderate complexity. Astrocytes in the MBmot showed the lowest complexity with minimal branching. By contrast, the STR and TH exhibited higher intersection counts, indicating substantial branching. Astrocytes in the VIS demonstrated a balanced profile with moderate complexity (**Figure 6B** and **C**). This detailed breakdown highlights region-specific differences in branching complexity, thus indicating the variability in astrocyte morphology.

Together, these variations in the Sholl analysis further emphasize the morphological diversity of astrocytes across different brain regions. Furthermore, this classic method of cell complexity analysis provides valuable insights into differences in astrocyte morphology, particularly in models of astrocyte-related diseases (Bird and Cuntz, 2019). The sparse labeling tool was highly effective for the extensive Sholl analysis of astrocytes, allowing for a comprehensive understanding of the structural diversity of these cells.

### Validation of the dual adeno-associated virus system for neuromodulation applications

**Figure 7:**
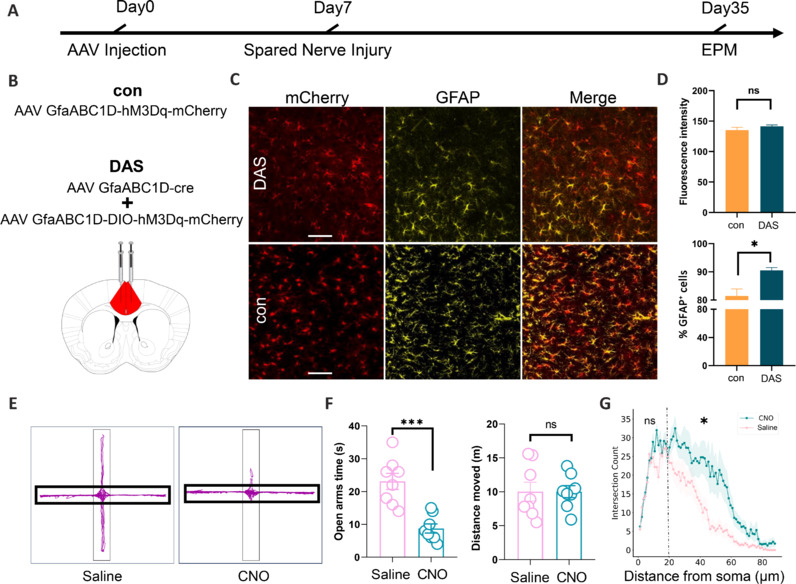
Validation of DAS technology for astrocyte neuromodulation applications in the SNI model. (A) Timeline of the experimental procedure. (B) Schematic diagrams of AAV injection into the anterior cingulate cortex, depicting the administration of GfaABC1D-hM3Dq-mCherry alone or DAS (Cre:DIO=1:1). (C) Immunofluorescent images of brain sections showing mCherry (red) and GFAP (yellow) expression in the con (upper) and DAS (lower) groups. Scale bars: 40 µm. (D) Quantification of the fluorescence intensities and percentages of GFAP-positive cells in the con and DAS groups (stereotaxic injection of the same dose of different vectors, *n* = 4). (E) Representative movement traces of mice treated with saline and CNO in the EPM. (F) Quantification of time spent in the open arms and distance moved in the EPM. (G) Sholl analysis curves for astrocytes from the saline (intraperitoneal injection of saline) and CNO (intraperitoneal injection of CNO) groups. Data were represented with mean ± SEM; *P < 0.05, ***P < 0.001 (two-sample *t*-test). AAV: Adeno-associated viruses; CNO: clozapine N-oxide; con: control; DAS: dual-AAV system; EPM: elevated plus maze; GFAP: glial fibrillary acidic protein; ns: not significant; SNI: spared nerve injury.

We tested various ratios of the DAS components and identified a stable combination with equal titers of both viruses, which effectively labeled multiple cells. Stereotaxic labeling across different brain regions using these ratios are shown in **Additional Figure 1**. To confirm the effectiveness of the DAS regulatory elements, we conducted a series of experiments outlined in the timeline in **Figure 7A**: AAV injection on day 0, spared nerve injury (SNI) on day 7, and the elevated plus maze test on day 35. A total of 100 L of viral solution was stereotaxically injected into the anterior cingulate cortex region of each mouse. The viral solution contained rAAV2/5-GfaABC1D-mCherry at a concentration of 1 × 10^8^ vg/mouse. Similarly, the rAAV2/5-GfaABC1D-DIO-mCherry virus and rAAV2/5-GfaABC1D-Cre virus were administered at the same dose (1 × 10^8^ vg/mouse) with a viral ratio of 1:1 (rAAV2/5-GfaABC1D-Cre to rAAV2/5-GfaABC1D-DIO-EGFP). **Figure 7B** illustrates the AAV injection procedure, with two experimental groups receiving either AAV GfaABC1D-hM3Dq-mCherry alone or using the DAS. Immunofluorescent images of brain sections labeled with mCherry and GFAP (**Figure 7C**) revealed substantial overlap in the merged images from both groups. Although quantification indicated no significant differences in fluorescence intensity between the control groups, the DAS group exhibited a significantly higher percentage of GFAP-positive cells (*P* < 0.05; **Figure 7D**), suggesting enhanced specificity for astrocyte labeling. In summary, by testing various ratios of DAS components, we identified a stable combination that effectively labeled multiple cells with equal titers of both viruses, thus demonstrating the versatility and efficiency of the DAS for stereotaxic labeling across different brain regions.

Next, we performed chemogenetic modulation on the DAS group. In the elevated plus maze test (**Figure 7E** and **F**), CNO-treated mice explored the open arms less than the saline-treated group, with significantly less time spent in the open arms (*P* < 0.001). By contrast, there was no significant difference in the total distance moved, indicating no change in overall locomotor activity (**Figure 7F**). In the Sholl analysis, the CNO group showed significantly different branching complexity compared with the control group (**Figure 7G**). These findings align with previous studies, indicating that our labeling strategy accurately validates the function of the loaded elements (Wei et al., 2024). Overall, our new labeling approach demonstrates both high specificity and robust functional performance for astrocytes, making it a valuable tool for neuroscience research.

## Discussion

In the present study, we established a DAS to sparsely label astrocytes in different brain regions in mice. We validated the labeling efficacy and specificity of this approach *in vivo* and explored the regional distributions of astrocyte morphology. An analysis of 772 individual cells revealed considerable morphological similarity across several brain regions, including the MO. A clustering analysis based on anatomical subdivisions highlighted differences in morphological parameters across regions. Notably, parameters such as graph density, which reflect more complex structural features, were more prominent in regions associated with higher-order cortical functions. Cortical astrocytes exhibit highly dynamic and region-specific morphologies that are essential for their functional roles in maintaining cortical homeostasis and supporting neuronal activity (Lanjakornsiripan et al., 2018). In cortical gray matter, astrocytes typically have a star-like shape with numerous, highly branched processes that extend to form complex networks, allowing them to interact with synapses, neurons, and blood vessels. These morphological features are closely linked to the diverse and critical functions that astrocytes perform in cortical areas (Sofroniew and Vinters, 2010). In the HIP, astrocytes typically have a stellate shape with extensive, highly branched processes that interact with both synapses and blood vessels (Batiuk et al., 2020). These morphological characteristics enable astrocytes to perform several critical functions that are essential for HIP function, particularly in learning and memory processes. Our study also examined the effects of chemogenetic manipulation of astrocytes in the anterior cingulate cortex on anxiety behaviors using a long-term SNI model. In this context, we observed increased branching in CNO-activated astrocytes, as confirmed by Sholl analysis. These findings contribute to our understanding of astrocyte morphology and its functional implications across different brain regions.

There are several approaches for sparsely and brightly labeling astrocytes in the brain. Most structural analyses of astrocytes have traditionally relied on GFAP, which is expressed in primary, secondary, and some tertiary branches of astrocytes but not in higher-order processes, thus excluding critical aspects of astrocyte morphology from examination. Another relatively straightforward method involves injecting dye into astrocytes within a specific brain region or subregion. This strategy enables the observation of morphological changes in astrocytes under both normal and pathological conditions (Zhou et al., 2019a; Baldwin et al., 2023). However, the efficiency of this method is relatively low, with a limited number of labeled cells and a short observation period, making it impractical for labeling astrocytes throughout the entire brain. Transgenic mice have also been used for astrocyte labeling; however, this method is constrained by lengthy breeding and growth cycles as well as species-specific limitations (Lu and Yang, 2017; Lanjakornsiripan et al., 2018). Viral-based methods for labeling astrocyte morphology, such as the use of constructs to study calcium signaling in astrocyte processes, have revealed valuable insights into astrocyte dynamics (Denizot et al., 2022). The use of Semliki Forest virus and rabies virus for short-term neuronal labeling has also provided an effective approach for sparsely labeling glial cells (Jia et al., 2019). Additionally, the application of directed evolution techniques has enabled the selection of serotypes for specific glial cell labeling, thus offering new avenues for sparse or specific labeling of these cells (Lin et al., 2022).

The DAS has been extensively studied in the context of neuronal labeling (Lu and Yang, 2017; Lin et al., 2018); this inspired us to apply the DAS to the specific labeling of astrocytes. Using the Cre-loxP system, we successfully achieved whole-brain astrocyte labeling across most mouse strains, demonstrating that this strategy is both mature and straightforward. A number of advantages were noted. First, by adjusting the viral titer, this strategy allows for controlled sparse and bright labeling as well as the manipulation of astrocytes. Second, the method of virus delivery is both simple and versatile, enabling the precise labeling of astrocytes in various brain regions via tail vein injection. This technique can also be adapted to use viruses capable of crossing the blood–brain barrier, or through the use of microinjections to target specific central or peripheral astrocytes. Third, the viral vector can carry not only fluorescent proteins for visualization but also additional components for manipulating astrocytes, thereby creating a dual-function system that combines both visualization and manipulation. Importantly, the morphological labeling remains unaffected by optogenetic or chemogenetic tools carried by the viruses. Moreover, this approach offers distinct advantages over other plasmid-based strategies because of its ease of design and relatively high efficiency. Lastly, the DAS achieves very good specificity of astrocyte labeling, making it a powerful tool for in-depth astrocyte research.

Astrocyte morphology holds potential for the study of regionally related diseases in brain regions (Zhou et al., 2019b). In data labeled across the blood–brain barrier, we analyzed astrocyte morphology in different regions of the brain. Moreover, the diversity of astrocyte morphology in different regions of the brain or within the same region was evident at relatively low Euclidean distance classification parameters. The analysis of morphological parameters provides valuable insights into astrocyte structure and function. Parameters such as process length, branching complexity, and process density reflect key physiological roles, such as synaptic modulation and response to injury. These metrics are also useful for identifying region-specific functions, as biomarkers in disease models, and for advanced imaging and labeling techniques. By highlighting their importance, these parameters contribute to a deeper understanding of astrocyte biology in both health and disease. With the optimization of classification parameters, we also observed the emergence of consistency across multiple brain regions, whereas heterogeneity remained in the SS and TH. These findings suggests that our morphological studies may need further refinement—for example, by increasing the sample size, employing more precise imaging techniques, using advanced image reconstruction methods, and extracting more accurate morphological parameters (Chen et al., 2021). Through the optimization of these methods, we may refine our morphological research down to the level of specific layers or subregions (Clavreul et al., 2019). Additionally, the use of *in vivo* imaging techniques will allow us to conduct long-term morphological studies of specific cells targeted by the vectors (Wu et al., 2022; Shekhtmeyster et al., 2023).

The current DAS faces several technical limitations that warrant future improvements. Although the GfaABC1D promoter enables astrocyte labeling, its expression extends beyond astrocytes, potentially affecting targeting specificity. Recent developments, such as the 4x6T micro RNA cassette in AAV vectors, show promise in addressing this issue by suppressing expression in non-target cells, achieving over 99% astrocyte specificity (Gleichman et al., 2023). The current analysis, which relied on fixed brain samples, provided detailed structural insights but lacked the dynamic perspective offered by *in vivo* imaging techniques. Incorporating these approaches would enable a more comprehensive understanding of astrocyte behavior in real-time. Additionally, to address the limitations of sample size, future studies will increase the numbers of labeled samples to improve the robustness and generalizability of findings, thereby allowing for more reliable group-level conclusions. Another key limitation of the current approach is the absence of integrated molecular analysis. Future studies should include molecular profiling techniques, such as transcriptomics or proteomics, to elucidate the molecular mechanisms underlying astrocyte morphology. Combining enhanced targeting specificity, dynamic imaging approaches, and integrated multi-omics analyses would offer a more holistic perspective (Endo et al., 2022). This comprehensive strategy may bridge the gap between structural observations and molecular insights, potentially identifying novel therapeutic targets and advancing our understanding of astrocyte function in both health and disease.

In conclusion, we developed a simple and straightforward strategy to perform sparse, cell-type-specific astrocyte labeling throughout the whole brain. Moreover, we used this strategy to investigate astrocyte morphology and manipulate its function in the anterior cingulate cortex of SNI model mice. Our results indicate that the DAS technology is promising for the sparse labeling of astrocytes and has improved astrocyte labeling efficiency compared with traditional technology. The ability to selectively label and study astrocytes in different brain regions provides a powerful tool for exploring the complexities of these essential cells and their roles in health and disease.

## Additional files:

***Additional Figure 1:*** *Validation of the DAS-based fluorescent labeling system across the blood–brain barrier in the whole mouse brain.*

Additional Figure 1 Validation of the DAS-based fluorescent labeling system across the blood–brain barrier in the whole mouse brain.
(A—C) Representative grayscale confocal images showing the distribution of sparsely labeled astrocytes across the blood–brain barrier in different brain regions of C57BL/6J mice; the numbers indicate the distance from bregma. (A) GfaABC1D-EGFP, (B) GfaABC1D-DIO-EGFP/GfaABC1D-Cre=1, and (C) GfaABC1D-DIO-EGFP/GfaABC1D-Cre=10. Scale bars: 2 mm. (D) Quantification of the average fluorescence intensity of each cell across various brain regions in the three groups. (E) Quantification of EGFP-positive areas across various brain regions in the three groups. Equal amounts of different viral vectors were injected into the tail vein of the three groups. DAS: Dual-AAV system; DIO/Cre: (rAAV/PHP.eB-GfaABC1D-DIO-EGFP)/(rAAV/PHP.eB-GfaABC1D-Cre); EGFP: enhanced green fluorescent protein. Data were represented with mean ± SEM, ^***^P < 0.001 (one-way analysis of variance with Bonferroni correction).

***Additional Figure 2:*** *Co-expression of NeuN and EGFP to exclude virus leakage under GFaABC1D promoters.*

Additional Figure 2 Co-expression of NeuN and EGFP to exclude virus leakage under GFaABC1D promoters.
(A) Immunofluorescent images of astrocytes labeled with NeuN (pink) and EGFP (green) in various CNS regions. Scale bars: 40 μm. (B) Percentage of NeuN-positive cells in each CNS region with DAS technology. Data were represented with mean ± SEM. ACA: Anterior cingulate area; AI: agranular insular cortex; CNS: central nervous system; DIO/Cre: (rAAV/PHP.eB-GfaABC1D-DIO-EGFP)/(rAAV/PHP.eB-GfaABC1D-Cre); EGFP: enhanced green fluorescent protein; FRP: frontal pole; HIP: hippocampus; MBmot: midbrain motor nucleus; MO: motor cortex; NeuN: neuron-specific nuclear protein; PIR: piriform cortex; RSP: retrosplenial cortex; SS: somatosensory cortex; STR: striatum; TH: thalamus; VIS: visual cortex.

***Additional Figure 3:*** *Astrocytes were subjected to two-dimensional pretreatment prior to morphological parameter analysis.*

Additional Figure 3 Astrocytes were subjected to two-dimensional pretreatment prior to morphological parameter analysis.
(A–D) Processing steps from grayscale images to binarized and skeletonized images for subsequent morphological quantification. Scale bars: 40 μm. EGFP: Enhanced green fluorescent protein.

***Additional Figure 4:*** *Schematic illustration demonstrating each morphological parameter.*

Additional Figure 4 Schematic illustration demonstrating each morphological parameter.
A simplified cell model illustrating the key morphological parameters used in the analysis of astrocytes, such as cell body size, territory, and network connectivity. These parameters are essential for quantifying the structural properties of astrocytes and advancing our understanding of their spatial distributions and organizational patterns.

## Data Availability

*All relevant data are within the paper and its Additional files*.
