# Supplementary figures and images for: Dual adeno-associated virus system for selective and sparse labeling of astrocytes
Source: Neural Regen Res. 2025 Jun 19;21(7):3083–91. doi: 10.4103/NRR.NRR-D-24-01607 (PMC13378953; doi:10.4103/NRR.NRR-D-24-01607)

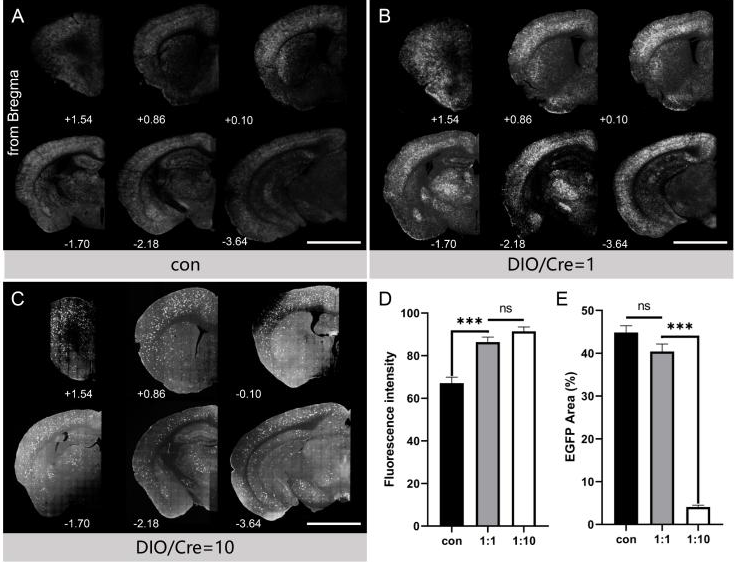

Supplement: Supplementary file 1 [file NRR-21-3083_Suppl1.tif]

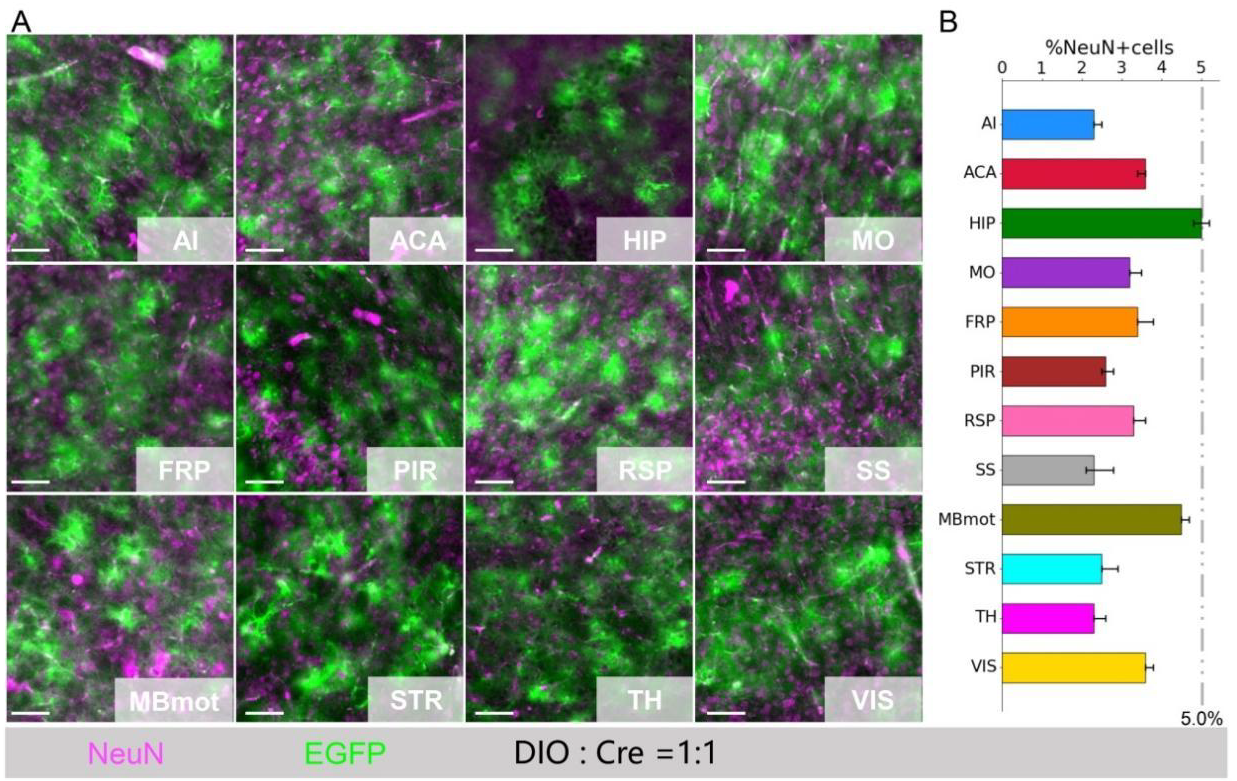

Supplement: Supplementary file 2 [file NRR-21-3083_Suppl2.tif]

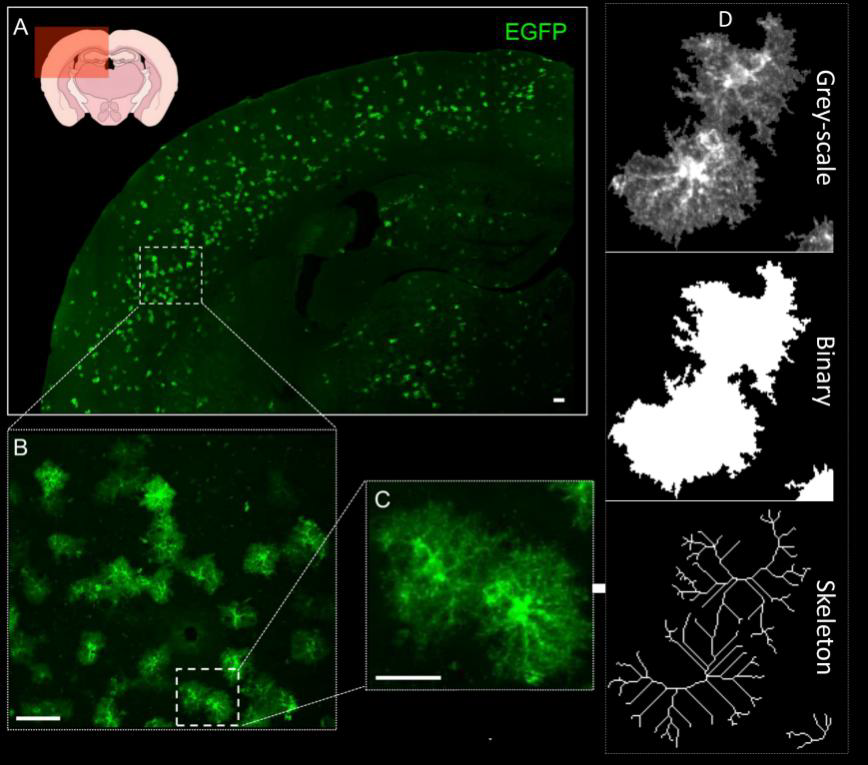

Supplement: Supplementary file 3 [file NRR-21-3083_Suppl3.tif]

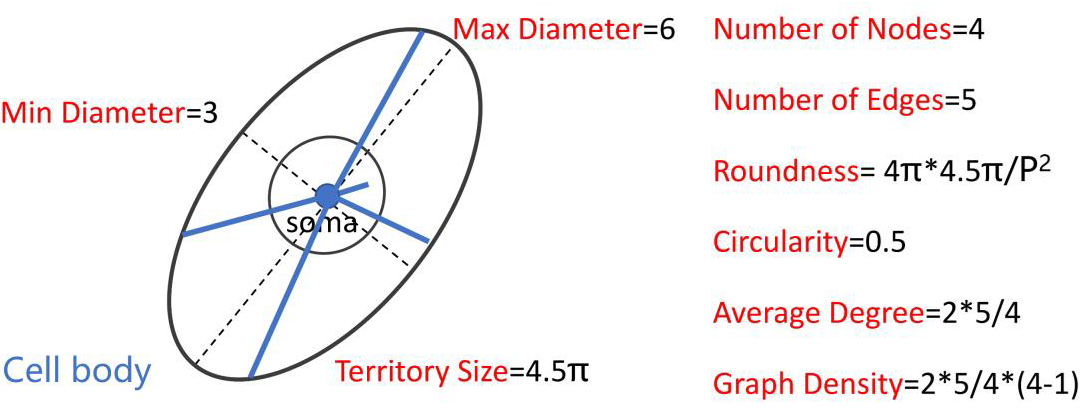

Supplement: Supplementary file 4 [file NRR-21-3083_Suppl4.tif]
